# Value of Pyruvate Carboxylase in Thyroid Fine-Needle Aspiration Wash-Out Fluid for Predicting Papillary Thyroid Cancer Lymph Node Metastasis

**DOI:** 10.3389/fonc.2021.643416

**Published:** 2021-05-17

**Authors:** Chang Liu, Lu Zhang, Yang Liu, Qingqing Zhao, Yu Pan, Yifan Zhang

**Affiliations:** ^1^ Department of Nuclear Medicine, Ruijin Hospital, Shanghai Jiao Tong University School of Medicine, Shanghai, China; ^2^ Department of Ultrasound, Ruijin Hospital, Shanghai Jiao Tong University School of Medicine, Shanghai, China

**Keywords:** papillary thyroid carcinoma, pyruvate carboxylase, biopsy, fine-needle, lymphatic metastasis

## Abstract

The incidence of papillary thyroid carcinoma (PTC) is increasing. Lymph node metastatic status of PTC is a major factor for decision marking of surgery and surgical extend, however, no reliable tool exists for prediction of PTC nodal metastasis, for example, ultrasound cannot qualitatively diagnose and effectively detect central lymph node metastasis (CLNM). Therefore, the development of a new diagnostic biomarker is crucial for CLNM. Metabolic dysregulation is an important factor associated with malignancy and metastasis of tumors. Pyruvate carboxylase (PC) is a major anaplerotic enzyme that catalyzes the carboxylation of pyruvate to form oxaloacetate, which has been suggested to be involved in the tumorigenesis of several cancers, including PTC. This study aimed to explore the role of *PC* expression in thyroid fine-needle aspiration (FNA) wash-out fluid for predicting CLNM in PTC, and to explore how PC is involved in PTC development. The expression levels of *PC* in PTC tissues and normal thyroid tissues were first compared based on bioinformatics analysis of public databases, including the Gene Expression Profiling (GEPIA), Oncomine and Gene Expression Omnibus (GEO) databases. Then, the *PC* mRNA and protein expression levels were measured by RT-PCR and Immunohistochemistry (IHC) in surgical tissues from a total of 42 patients with surgically confirmed PTC, and compared in patients with and without CLNM. Further, to assess *PC* expression in diagnostic biopsies, a total of 71 thyroid nodule patients with ultrasound-guided FNA wash-out fluid samples and cytological diagnosis were prospectively enrolled in the study. Then, we analyzed the mechanism of PC-mediated PTC progression *in vitro*. This study showed that PC expression was higher in PTC tissues and thyroid FNA wash-out fluid samples from patients with CLNM than those from patients without CLNM, and that PC-induced PTC metastasis may occur through the TGF-β/Smad-regulated epithelial–mesenchymal transition (EMT) pathway.

## Introduction

Thyroid carcinoma is the most common endocrine tumor in the world, and its worldwide incidence has significantly increased recently (1, 2). Nearly 90% of thyroid cancers (THCAs), including papillary thyroid carcinoma (PTC) and follicular thyroid carcinoma (FTC), are well differentiated (3). PTC is the most common subtype, accounting for 80–85% of all malignant thyroid tumors (2). PTCs exhibit indolent biological behavior (4), and the 5-year survival rate reaches almost 95% after treatment (5), including surgery and radioactive iodine therapy. There is a debate about whether or not PTC, especially papillary thyroid microcarcinoma (PTMC), is overdiagnosed and overtreated. Ito et al. (6) reported that only a small portion of PTMCs showed progression and suggested that subclinical low-risk PTMC should be surveilled instead of treated by immediate surgery. On the other hand, some PTMCs show nodular enlargement and early lymph node metastasis during the surveillance period (7). Thus, it is critical to develop a tool to identify patients with early nodal metastasis to guide clinical management.

Cancer cell proliferation requires energy, which is dependent on cellular metabolism (8). In proliferating cancer cells, the replenishment of intermediates for the tricarboxylic acid (TCA) cycle provides biosynthetic precursors for the synthesis of proteins, nucleic acids and lipids (9, 10). The pyruvate carboxylase (PC)-mediated anaplerosis pathway for TCA intermediates is implicated in the progression of a variety of cancers. For example, compared with that in non-small-cell lung cancer (NSCLC) without metastasis, the expression of PC is increased in NSCLC with metastasis (11, 12). In the early stages of NSCLC, PC knockdown can significantly increase TCA activity and inhibit biosynthesis (12). It has also been reported that, compared to that in primary tumors, PC-catalyzed generation of oxaloacetate occurs at a higher rate in breast cancer with lung metastasis (13–15). Chen et al. (16)found that a small molecule ZY444, which binds to PC, can significantly inhibit breast cancer growth and metastasis *in vitro* and *in vivo*. In particular, Strickaert et al. reported that PC-mediated replenishment of the TCA cycle plays a leading role in PTC and knockdown PC influence thyroid cancer cells progression abilities (17). However, it is unknown how PC affects the clinical manifestations of THCA and what is the underlying mechanism.

At present, the main clinical diagnostic methods of PTC are ultrasonography and fine-needle aspiration biopsy (FNAB) (18). However, given the inherent limitations of current diagnostic methods, it is difficult to identify central lymph node metastasis (CLNM) located in the neck, particularly the areas surrounded by the hyoid bone and the sternal notch (19), the initial sites of lymph node metastasis (20). With the development of basic medical research, molecular genetic alterations have been increasingly studied, and many tumor biomarkers have been found, improving the accuracy of PTC risk categories (4). The most widely studied and clinically used PTC tumor markers are BRAF and TERT promoter mutations (21). Although the incidences of the two mutations are 83.7 and 7.5–27%, respectively (22, 23), neither has been shown significant prognostic value for predicting the high-risk clinicopathological features of PTC (24). Therefore, it is important to explore other tumor markers that could potentially predict the aggressive biological behavior of PTC, including nodal metastasis. The study aimed to test the hypothesis that *PC* expression in the thyroid nodule FNA wash-out fluid can potentially predict thyroid cancer regional nodal metastasis as diagnosed after surgery, and further to explore the underlying mechanism.

## Materials and Methods

### Gene Expression Data Acquisition and Bioinformatics Analysis

Gene Expression Profiling Interactive Analysis (GEPIA)-(http://gepia.cancer-pku.cn/) (25) is an online server for the profiling and interactive analyses of gene expression in cancer and normal samples. Oncomine (https://www.oncomine.org/) (26, 27) is an online cancer microarray database and integrated data-mining platform. We searched PC in the two databases to analyze *PC* expression in THCA and other types of cancer. In addition, we used expression profiling arrays (GSE60542) from Gene Expression Omnibus databases to analyze the differentially expressed genes between THCA tissues from patients with or without lymph metastasis. The GSE60542 dataset was analyzed by the Affymetrix Human Genome Array with the annotation platform GPL570 [HG-U133_plus_2]. The GSE60542 dataset contained 14 PTC samples from patients with no lymph node metastasis, 19 samples from patients with lymph node invasion and 10 control samples from patients with normal thyroids. Using R packages, we assessed the raw GSE60542 dataset by background correction, normalization, expression calculation, and probe integration. Robust multiarray averages (RMAs) and mismatch probes (PMs) were created for processing the datasets. *P*-values were adjusted by the Benjamini–Hochberg method, and fold changes (FC) were calculated using the false discovery rate (FDR) procedure. Differentially expressed genes (DEGs) with a |log2-fold change| >1 and *P <*0.05 in PTC samples versus normal samples were selected. The basic features from the Gene Expression Omnibus (GEO) database are shown in **Table 1**.

**Table 1:** Basic features of the GSE 60542 database.

GEO no.	Subtype
GSM 1481838	Normal thyroid
GSM 1481842	Normal thyroid
GSM 1481852	Normal thyroid
GSM 1481862	Normal thyroid
GSM 1481877	Normal thyroid
GSM 1481891	Normal thyroid
GSM 1481895	Normal thyroid
GSM 1481901	Normal thyroid
GSM 1481907	Normal thyroid
GSM 1481913	Normal thyroid
GSM 1481844	Papillary thyroid carcinoma, N1
GSM 1481890	Papillary thyroid carcinoma, N1
GSM 1481847	Papillary thyroid carcinoma, N1
GSM 1481875	Papillary thyroid carcinoma, N1
GSM 1481929	Papillary thyroid carcinoma, N1
GSM 1481854	Papillary thyroid carcinoma, N1
GSM 1481905	Papillary thyroid carcinoma, N1
GSM 1481861	Papillary thyroid carcinoma, N1
GSM 1481882	Papillary thyroid carcinoma, N1
GSM 1481849	Papillary thyroid carcinoma, N1
GSM 1481865	Papillary thyroid carcinoma, N1
GSM 1481883	Papillary thyroid carcinoma, N1
GSM 1481903	Papillary thyroid carcinoma, N1
GSM 1481915	Papillary thyroid carcinoma, N1
GSM 1481839	Papillary thyroid carcinoma, N1
GSM 1481872	Papillary thyroid carcinoma, N1
GSM 1481926	Papillary thyroid carcinoma, N1
GSM 1481889	Papillary thyroid carcinoma, N1
GSM 1481914	Papillary thyroid carcinoma, N1

### Patient Populations With Surgically Confirmed PTC

The study enrolled 42 patients with surgically confirmed PTC and available surgical tissues from July to November 2018. The clinical characteristics of the patients are shown in **Table 2**.

**Table 2:** Clinicopathological characteristics of 42 surgically confirmed PTC patients.

Clinicopathological characteristics		Value	PC expression		*P*
			High	Low	
Gender					
male		14	5	9	0.491
female		28	7	21	
Age (Y), Mean ± SD		42.3 ± 1.9			
≥55, n (%)		8 (19.0%)	3	5	0.668
<55, n (%)		34 (81.0%)	9	25	
Tumor size (mm)	≥5, n (%)	33 (78.6%)	11	27	1
	<5, n (%)	9 (21.4%)	1	3	
CLNM	Positive	15 (35.7%)	11	16	0.031*
	Negative	27 (64.3%)	1	14	
Extrathyroidal extension	Positive	27 (64.3%)	6	9	0.292
	Negative	15 (35.7%)	6	21	
Multifocal tumor	Positive	14 (33.3%)	6	8	0.169
	Negative	28 (66.7%)	6	22	
Bilateral distribution	Positive	21 (50%)	7	14	0.734
	Negative	21 (50%)	5	16	
TNM stage	I + II	29 (69%)	8	21	1
	III + IV	13 (31%)	4	9	

PTC, papillary thyroid carcinoma; CLNM, central lymph nodal metastasis; PC, pyruvate carboxylase, TNM, tumor-node-metastasis. *P <0.05.

### Patient Populations With Thyroid Nodules

A total of 71 patients with thyroid nodules and FNA wash-out fluid samples from November 2019 to January 2020 were also included. Fifty-two of the 71 patients were diagnosed with PTC, and 19 had benign nodules based on cytological findings. Thirty-four of the 52 cytologically diagnosed PTC patients underwent total thyroidectomy and central nodal dissection with final tissue sample confirmation. The clinical characteristics of the patients are shown in **Table 3**.

**Table 3:** Clinicopathological characteristics of 71 thyroid nodule patients.

Clinicopathological characteristics		Value	PC expression		*P*
			High	Low	
Gender					
male		27	11	16	0.807
female		44	20	24	
Age (Y), Mean ± SD		44.5 ± 1.5			
≥55, n (%)		20 (28.3%)	11	15	1
<55, n (%)		51 (71.8%)	20	25	
Cytology diagnosis (71)					
PTC		52 (73.2%)	28	24	0.006*
Benign nodule		19 (26.3%)	3	16	
Surgically diagnosed PTC (34)					
CLNM	Positive	17 (50.0%)	11	6	0.037*
	Negative	17 (50.0%)	4	12	
Tumor size (mm)	≥5, n (%)	25 (73.5%)	11	14	1
	<5, n (%)	9 (26.5%)	4	4	
Extrathyroidal extension	Positive	16 (47.1%)	7	9	
	Negative	18 (52.9%)	8	9	1
Multifocal tumor	Positive	27 (79.4%)	12	15	1
	Negative	7 (20.6)	3	3	
Bilateral distribution	Positive	13 (38.2%)	7	9	1
	Negative	21 (61.8%)	8	9	
TNM stage	I + II	28 (82.4%)	14	13	0.186
	III + IV	6 (17.6%)	1	5	

PTC, papillary thyroid carcinoma; CLNM, central lymph nodal metastasis; PC, pyruvate carboxylase; TNM, tumor-node-metastasis. *P <0.05.

### Sample Preparation

Surgical PTC tissues were divided into two parts: one part was fixed in 4% paraformaldehyde, and the other part was preserved in RNA protector and frozen at −80°C. For ultrasound-guided FNA, three passes were performed for each thyroid nodule, and direct smears were prepared from each pass for hematoxylin and eosin (HE) staining after air drying. Thyroid FNA wash-out fluid with RNA protector was collected from needles. According to the 8th Edition of the American Joint Committee on Cancer (AJCC) TNM staging Systems for thyroid cancer, we divided all surgical PTC patients into early stage I or II, and later stage III or IV.

### Quantitative Real-Time Reverse Transcription PCR

RNA was extracted from PTC tissues using TRIzol (Sangon Biotech, Shanghai, China). RNA of the thyroid nodule FNA wash-out fluid samples was extracted following the RNA kit protocol (Qiagen Hilden, Germany). Total RNA was first reverse-transcribed into cDNA using PrimeScript™ RT Master Mix (TaKaRa Bio Inc., Japan) (37°C for 15 min, 85°C for 5 s, and cooled to 4°C). RT-PCR of *PC* and the reference gene *β-actin* was performed following the protocol for TB Green^®^ Premix Ex Taq™ II (TaKaRa Bio Inc., Japan) in an Applied Biosystems 7500 Real-Time PCR System. The temperature cycling protocol consisted of 30 sec denaturation at 95°C, followed by 40 cycles of 95°C for 5 s and 60°C for 34 s. The 40 cycles were followed by 95°C for 15 s, 60°C for 1 min, and 95°C for 15 s. The *PC* primers used in this study were 5’-ATGTTGCCCACAACTTCAGCAAGC-3’ (forward primer) and 5’-AGTTGAGGGAGTCAAACACACGGA-3’ (reverse primer). The *TGF-βR1* primers used in this study were 5’-GTGACAGATGGGCTCTGCTT-3’ (forward primer) and 5’-AGGGCCAGTAGTTGGAAGTT-3’ (reverse primer). The *β-actin* primers were 5’-GCACCACACCTTCTACAATG-3’ (forward primer) and 5’-TGCTTGCTGATCCACATCTG-3’ (reverse primer). The *PC* mRNA expression level was normalized to that of *β-actin*. Cycle threshold (Ct) values below 35 were used in this study. The 2^−Δ Ct^ of *PC* mRNA–*β-actin* mRNA was used to evaluate the expression levels of *PC*.

### Immunohistochemistry (IHC)

Forty-two PTC tissues were soaked in 4% paraformaldehyde, embedded in paraffin, and cut into 4 μm thick slices. The staining intensity was evaluated by two pathologists who did not know the patient’s condition. Assess the percentage of positive cells by score: <10%, 0; 10–25%, 1; 26–50%, 2; 51–75%, 3; and 75%, 4. Assess the intensity of positive cells by score: 0 (negative), 1 (weak), 2 (moderate), 3 (moderately strong), or 4 (strong). The percentage of positive cells multiplied by the intensity score is the final score: <2, (−); ≥2–<7, (+); ≥7–<12, (++); ≥12 (+++).

### Cell Culture and Transfections

The human PTC cell line TPC1 was cultured in RPMI-1640 medium (Gibco, Thermo Scientific, MA, USA) containing 10% fetal bovine serum (FBS) (Gibco, Thermo Scientific, MA, USA) and 1 penicillin in 37°C and 5% CO_2_ culture. A lentiviral vector containing PC short hairpin RNA (shRNA), TGF-βR1 cDNA and its negative control was purchased from Hanbio (Shanghai, China) and transfected into TPC1 cells. Stable lentivirus-infected TPC1 cells were cultured in complete medium and screened with 1 µg/ml puromycin.

### Western Blotting

The cells were lysed with RIPA buffer (Epizyme, Shanghai, China) for 30 min on ice. The protein concentration was measured using a BCA protein assay kit (Epizyme, Shanghai, China). The protein was loaded onto a 4–20% SDS-PAGE gradient gel (Tanon, Shanghai, China) and then transferred to a polyvinylidene fluoride (PVDF) membrane (Millipore, Michigan, USA). After blocking, the membrane was incubated with the primary antibody overnight at 4°C and then washed three times with 1 × TBST (Tris buffered saline and Tween 20), and then the secondary antibody was incubated for 1 h. Finally, the membrane was rinsed with 1 × TBST three times and detected by an enhanced chemiluminescence kit (Tanon, Shanghai, China). The antibodies used were PC (Santa Cruz Biotechnology, TX, USA), Snail1 (Affinity Biosciences, OH, USA), ZEB1 (Affinity Biosciences, OH, USA), phospho-Smad2/3 (Cell Signaling Technology, MA, USA), Smad2/3 (Cell Signaling Technology, MA, USA), TGF-βR1 (Affinity Biosciences, OH, USA), E-cadherin (Affinity Biosciences, OH, USA), Vimentin (Affinity Biosciences, OH, USA), and GAPDH (BBI Solutions, Shanghai, China).

### Cell Counting Kit (CCK-8) Assay

The cells were cultured on a 96-well plate at a density of 1 × 10^4^ cells/well. Twenty-four hours later, 10 μl of CCK8 reagent was added to each well and incubated for 4 h at 37°C with 5% CO_2_. Then, a microplate reader was used to read the absorbance at 450 nm.

### Wound Healing

Wound healing was used to determine the migration ability of cells. Cells were seeded in a six-well plate at a density of 1 × 10^6^/ml and cultured at 37°C with 5% CO_2_ to a cell density of 100%. A 200 μl pipette tip was used to make a straight line and incubated for 48 h. The width of the scratch was observed under a microscope (Carl Zeiss, Germany).

### Transwell Assay

A total of 3 × 105 cells were suspended in 500 μl serum-free medium and added to the upper chamber of a Transwell plate (Costar, USA). Then, 750 μl of 20% FBS-containing medium was added to the lower chamber. The culture was continued for 48 h, and the cells were fixed with 4% paraformaldehyde and stained with 0.1% crystal violet. The invaded cells were counted with a microscope (Carl Zeiss, Germany).

### Statistical Analyses

Unless otherwise indicated, the data are expressed as the median [interquartile range]. The mRNA expression levels of *PC* in patients with cytologically diagnosed PTC were compared to those in patients with benign nodules using the Mann–Whitney *U* test. The chi-square test analyses for the association between clinicopathologic factors and *PC* expression level of surgically diagnosed PTC patients. The binary logistic regression test was used for multivariate analysis. Unpaired t tests were used for comparisons between two groups. All statistical analyses were conducted using SPSS 22.0. For all analyses, *P <*0.05 was considered statistically significant.

## Results

### Online Dataset Analysis of PC Expression in THCA

**Figure 1:**
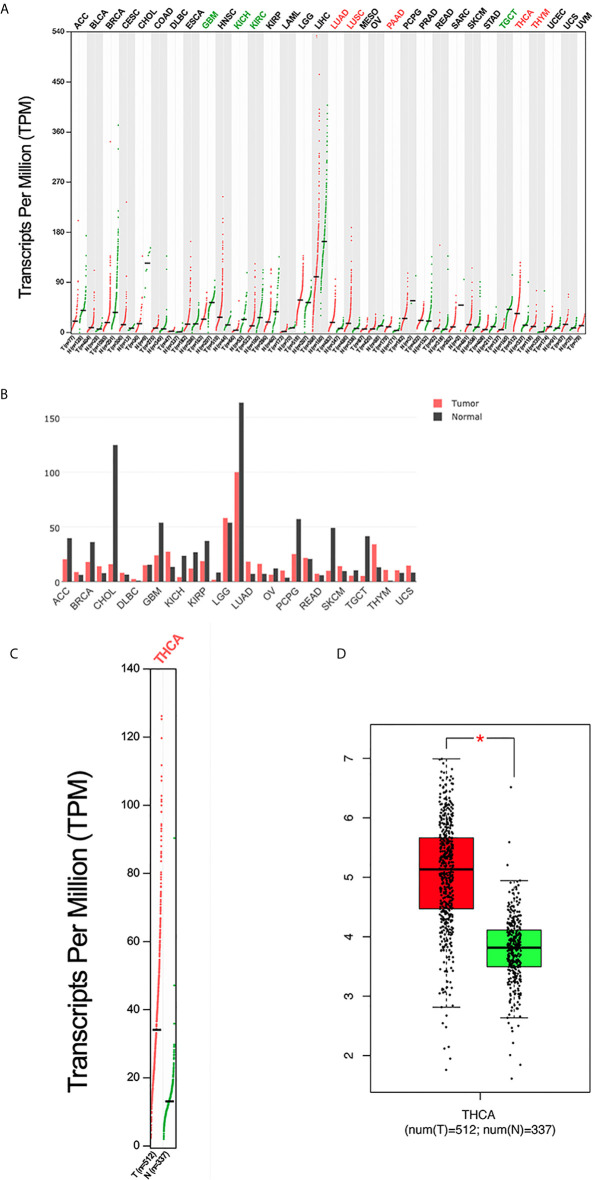
The expression profile of PC according to the GEPIA database. **(A)** The expression of PC in all tumor samples and paired normal tissues (dot plot). Each dot represents the expression of a sample. **(B)** The expression of PC in all tumor samples and paired normal tissues (bar plot). The height of bar represents the median expression of certain tumor type or normal tissue. **(C)** The expression of PC in THCA (dot plot); |log_2_FC| = 1 and *P <*0.01. **(D)** The expression of PC in THCA (box plot); |log_2_FC| = 1 and *P < 0.01. GEPIA, Gene Expression Profiling Interactive Analysis; T, tumor; N, normal; ACC, adrenocortical carcinoma; BLCA, bladder urothelial carcinoma; BRCA, breast invasive carcinoma; CESC, cervical squamous cell carcinoma and endocervical adenocarcinoma; CHOL, cholangiocarcinoma; COAD, colon adenocarcinoma; DLBC, lymphoid neoplasm diffuse large B-cell lymphoma; ESCA, esophageal carcinoma; GBM, glioblastoma multiforme; HNSC, head and neck squamous cell carcinoma; KICH, kidney chromophobe; KIRC, kidney renal clear cell carcinoma; KIRP, kidney renal papillary cell carcinoma; LAML, acute myeloid leukemia; LGG, brain lower grade glioma; LIHC, liver hepatocellular carcinoma; LUAD, lung adenocarcinoma; LUSC, lung squamous cell carcinoma; MESO, mesothelioma; OV, ovarian serous cystadenocarcinoma; PAAD, pancreatic adenocarcinoma; PCPG, pheochromocytoma and paraganglioma; PRAD, prostate adenocarcinoma; READ, rectum adenocarcinoma; SARC, sarcoma; SKCM, skin cutaneous melanoma; STAD, stomach adenocarcinoma; TGCT, testicular germ cell tumor; THCA, thyroid carcinoma; THYM, thymoma; UCEC, uterine corpus endometrial carcinoma; UCS, uterine carcinosarcoma; UVM, uveal melanoma.

**Figure 2:**
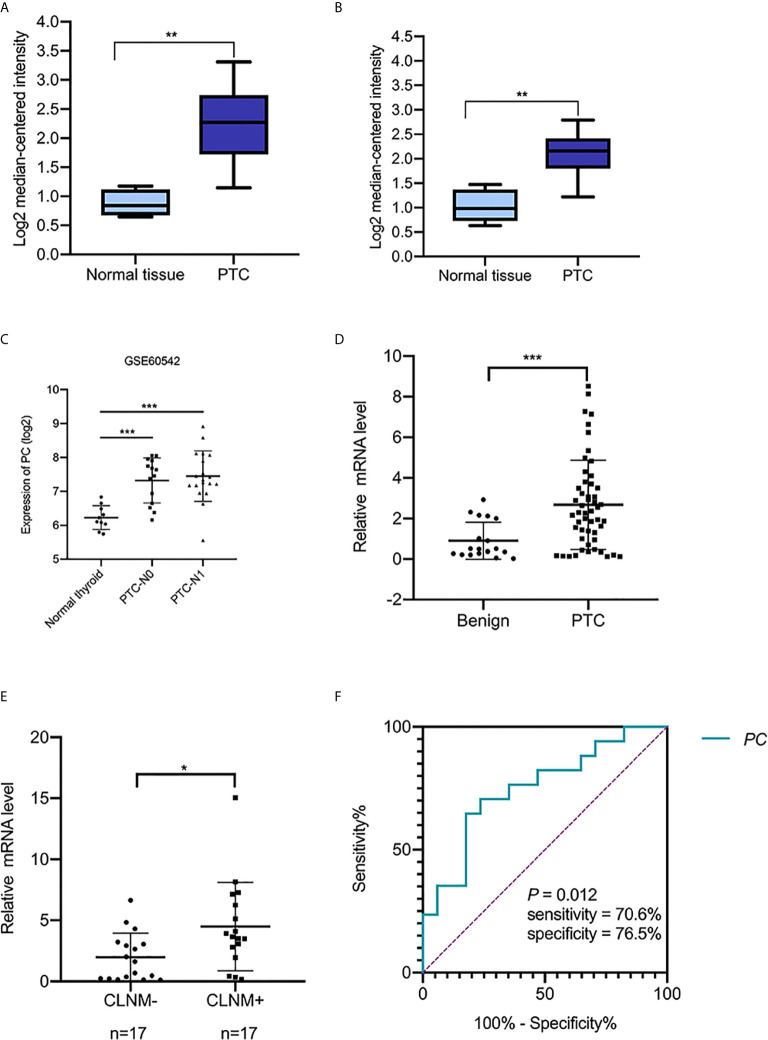
The transcript levels of PC in the Oncomine and GEO datasets and RT-PCR analysis of PC mRNA expression levels in thyroid FNA wash-out fluid. **(A)** PC level in the THCA dataset (Vasko’s dataset), (^**^ *P <*0.01). **(B)** PC levels in the THCA dataset (He’s dataset), (^**^ *P <*0.01). **(C)** PC levels in THCA dataset (GSE60542). ***P <0.001 **(D)** The expression of PC in 52 PTC FNA wash-out fluid samples and 19 benign nodule FNA wash-out fluid samples based on cytological diagnosis was examined using RT-PCR assays. **P <0.01. **(E)** Expression of PC in 34 surgically diagnosed PTCs from patients with or without CLNM. *P <0.05. **(F)** AUC for identifying PTC with CLNM using the PC mRNA expression level. The AUC was 0.751 (*P* = 0.012; 95% CI, 0.585–0.917). AUC, area under the receiver operating characteristic curve; PC, pyruvate carboxylase; PTC, papillary thyroid carcinoma; PTC-N0, papillary thyroid carcinoma without lymph metastasis; PTC-N1, papillary thyroid carcinoma with lymph metastasis; CLNM, central lymph node metastasis; CI, confidence interval.

*PC* mRNA expression in the GEPIA datasets differed in different types of tumors (**Figures 1A, B**) but was significantly overexpressed in THCA (**Figures 1C, D**). In addition, the expression of *PC* was also significantly higher in PTC than in normal tissues in two separate public datasets in Oncomine. In Vasko’s dataset, *PC* was overexpressed in THCA samples versus normal samples with a fold change of 2.516 (**Figure 2A**). In He’s dataset, *PC* was also highly expressed with a fold change of 2.061 (**Figure 2B**). According to GSE60542, the *PC* expression level was significantly higher in PTC samples from patients with or without lymph metastasis tissues than in normal thyroid tissues, and the fold changes were 1.10 and 1.22, respectively (**Figure 2C**).

### The Difference of PC mRNA Expression in PTC Tumor Tissues From Patients With or Without CLNM

To confirm the *PC* mRNA overexpression in THCA with lymph metastasis, as observed in the above online datasets, we measured the *PC* mRNA levels in surgical tissues of 42 surgically diagnosed PTC patients to further assess the relationship between PC and CLNM. There were 15 PTC patients without CLNM and 27 patients with CLNM. *PC* mRNA expression was higher in CLNM-positive patients than in CLNM-negative patients [6.490 (2.351–10.002) *vs*. 2.430 (1.466–4.976); *P* = 0.014]. Based on the median value of *PC* mRNA expression, the samples were divided into *PC* high level group and low level group. Chi-square test showed that high level group of *PC* had more CLNM (*P* = 0.031). Further analysis showed there is no difference between *PC* level and other factors, such as gender (*P* = 0.491), age (*P* = 0.668), extrathyroidal extension (*P* = 0.292), multifocal (*P* = 0.169), bilateral distribution (*P* = 0.734), and TNM staging (*P* = 1) (**Table 2**).

### PC Expression and Patient Age Were Independent Predictors of PTC With CLNM After FNA

The abovementioned factors were measured in surgical tissues. To investigate the nodal metastasis predictive role of PC from the preoperative diagnostic ultrasound-guided FNA fluids, *PC* expression levels were measured in 71 thyroid nodule FNA wash-out fluid samples. The 71 patients were divided into two groups according to their cytological diagnosis: 52 had PTC, and 19 had benign thyroid diseases. A Mann–Whitney *U* test showed that *PC* expression levels were significantly higher in those with PTCs than in those with benign nodules [2.456 (0.442–3.779) *vs*. 0.498 (0.262–2.010), *P* = 0.005; **Figure 2D**], consistent with the public dataset results described above. Additional, Chi-square test showed that high level group of *PC* associated with high PTC (*P* = 0.006). But there is no difference between *PC* level and gender (*P* = 0.807) and age (*P* = 1) (**Table 3**).

Thirty-four out of the 52 PTC patients underwent total thyroidectomy and nodal dissection with a final diagnosis of nodal metastasis status. *PC* expression level was found higher in the CLNM-positive group than in the CLNM-negative group [3.665 (2.378–6.691) *vs*. 1.621 (0.228–3.144), *P* = 0.013; **Figure 2E**]. Univariable logistic regression analysis revealed that PC expression and ages <55 were associated with CLNM (all *P <*0.05), but extrathyroid invasion, multiple-site invasion, bilateral invasion were not associated with CLNM. Multivariable analysis confirmed that PC and younger age were independent predictors of CLNM (**Table 4**).

**Table 4:** Univariable and multivariate analyses of predictive factors for CLNM in patients with PTC after FNA.

Variable		Univariate analysis			Multivariate analysis		
		*P*	OR	95% CI	*P*	OR	95% CI
Gender	male	0.493	1.607	0.414–6.240			
	female		1				
Ages (years)	<55	0.034*	6.667	1.151–38.598	0.045*	37.665	1.088–1304.1
	≥55		1			1	
Tumor size (cm^3^)	<5	0.698	1.354	0.293–6.261			
	≥5						
PC		0.029*	1.472	1.04–2.085	0.039*	1.728	1.028 – 2.906
Extrathyroidal extension	Positive	0.174	2.619	0.655–10.478			
	Negative		1				
Multifocal tumor	Positive	0.216	0.320	0.053–1.949			
	Negative		1				
Bilateral distribution	Positive	0.724	1.283	0.321–5.134			
	Negative						
TNM stage	I + II	0.102	6.667	0.684–64.772			
	III + IV		0.2				

CLNM, central lymph nodal metastasis; PC, pyruvate carboxylase; CI, confidence interval; OR, odds ratio; TNM, tumor-node-metastasis. *P <0.05.

To test the sensitivity and specificity of PC expression as a biomarker for PTC with CLNM, we applied ROC curve analysis. The area under the curve (AUC) was 0.751 (*P* = 0.013, **Figure 2F**). The sensitivity and specificity were 70.6 and 76.5%, respectively. The results suggest that PC expression is potentially a novel biomarker for CLNM of PTC.

### The Difference of PC Protein Expression in PTC Tumor Tissues From Patients With or Without CLNM

**Figure 3:**
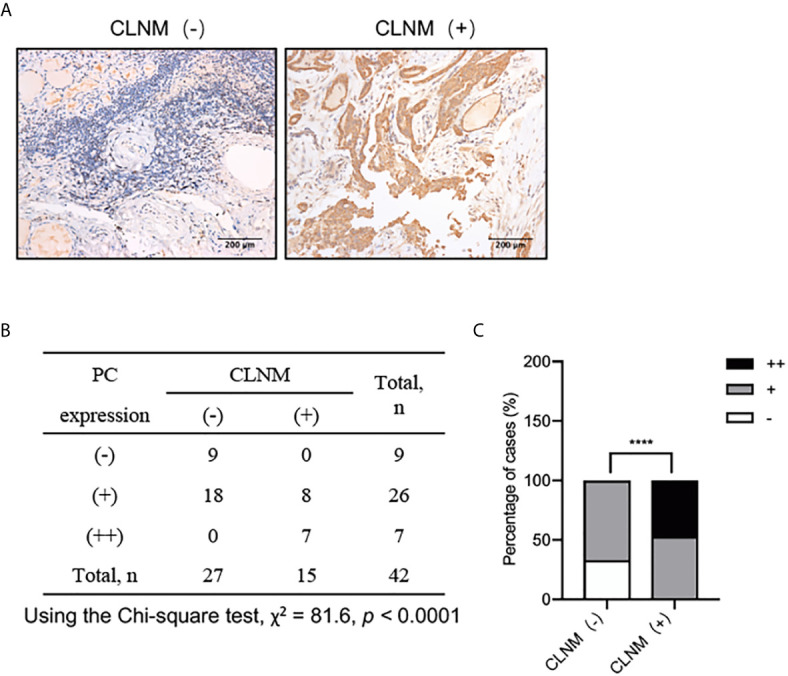
IHC analysis of PC protein expression levels in 42 surgically diagnosed PTCs with or without CLNM. **(A)** Representative IHC images of PTC samples form patients with or without CLNM. **(B)** Chi-square analysis of the protein expression levels of PC between PTC samples from patients with CLNM and PTC samples from patients without CLNM, as show in the histogram plot. **(C)** ****P <0.0001. CLNM, central lymph nodal metastasis; IHC, immunohistochemistry.

The above results suggest that PC mRNA may be related to CLNM of PTC, so we further investigated the relationship between the protein expression of PC in PTC tissues from patients with or without CLNM by IHC. The PC protein expression levels were significantly higher in CLNM-positive patients than in CLNM-negative patients (*P <*0.0001) (**Figures 3A–C**): out of the 15 CLNM positive samples, eight (53%) were (+) and seven (47%) were (++), while, of the 27 CLNM negative samples, nine (33%) were (−) and 18 (67%) were (+). The increased expression of both the PC mRNA and protein in the tissues with CLNM suggests that PC may play an important role in the malignant biological behavior of PTC, but the specific mechanism is still unclear.

### PC Promotes TPC1 Cell Migration and Invasion

**Figure 4:**
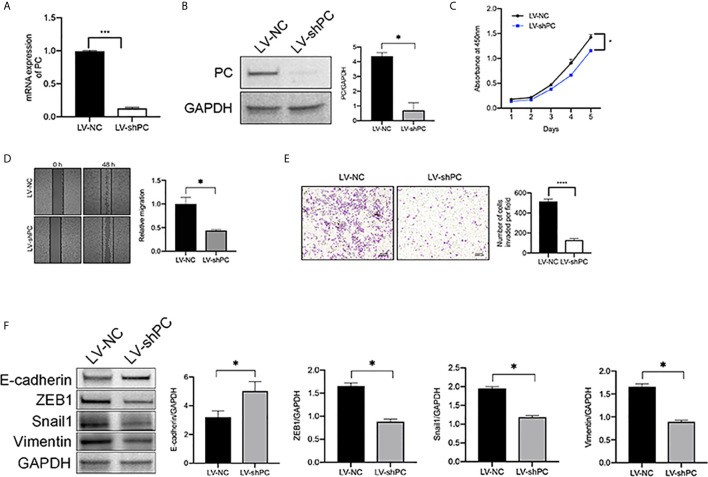
PC knockdown inhibit the migration and invasion of PTC cells. **(A)** qRT-PCR analysis of *PC* mRNA expression in the PTC cell line (TPC1) as indicated. ***P <0.001 **(B)** Western blotting was used to evaluate PC protein expression in TPC1 cells as indicated. The average quantification obtained by densitometric analysis (Image J) of the blot of PC expression. Data are expressed as mean ± SEM. *P <0.05. **(C)** CCK-8 assay was used to detect the proliferation of the indicated TPC1 cells. *P <0.05. **(D)** A wound-healing assay was applied to assess the migration ability of different TPC1 cells (LV-NC, LV-shPC). Representative images and quantification of cell counts normalized by controls are shown. *P <0.05 **(E)** Transwell invasion assays were applied to assess the motility of different TPC1 cells with a 4× objective lens. ****P <0.0001. The number of invaded cells is shown in histograms on the right. **(F)** Western blot analysis of E-cadherin, Snail1, Vimentin, and ZEB1 expression in LV-NC cells compared with LV-shPC cells. The average quantification obtained by densitometric analysis (Image J) of the blot of proteins expression. Data are expressed as mean ± SEM. *P <0.05. The full blot is provided in the **Supplementary File**.

To analysis the mechanism of PC in THCA cell aggressiveness, we first constructed transgenic TPC1 cells stably expressing PC knockdown lentivirus (LV-shPC) and a negative control lentivirus (LV-NC) (**Figures 4A, B**). CCK8, wound healing, and Transwell assays showed that the proliferation, migration, and invasion ability were significantly decreased in LV-shPC cells compared to LV-NC cells (**Figures 4C–E**).

PTC is a kind of tumor originating form follicular epithelial cells (28–31). Epithelial origin tumors are largely dependent on epithelial–mesenchymal transition (EMT) for metastasis (32, 33). Thus, we explored whether PC is related to EMT activation in PTC at cellular level. Consistently with our hypothesis, western blot assay results showed that the protein levels of EMT transcription factors (34), such as zinc finger E-box-binding homenbox1 (ZEB1), Snail1, and Vimentin, were decreased and that the protein levels of E-cadherin was increased in LV-shPC cells compared to LV-NC cells (**Figure 4F**). The results suggest that PC is related to PTC aggression.

### PC Induces EMT in TPC1 Cells *via* the TGF-β/Smad Pathway

It has been reported that, EMT can be activated by transforming growth factor beta 1 (TGF-β1) signaling through the Smad pathway (35–38). We hypothesized that PC induces TPC1 cell proliferation, migration, and invasion through the TGF-β/Smad pathway.

**Figure 5:**
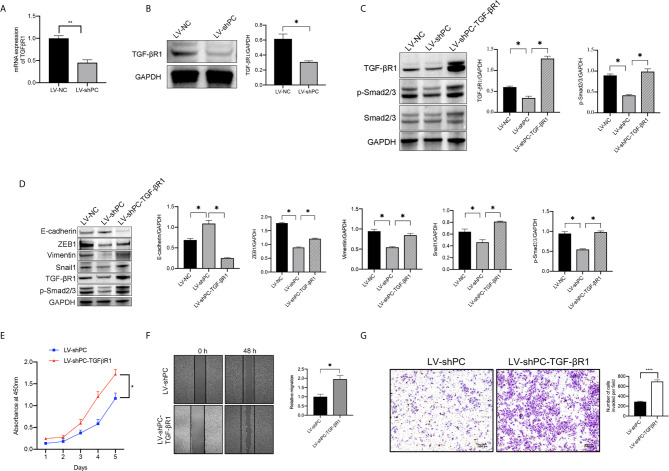
PC induces EMT in PTC cells by activating the TGF-β/Smad signaling pathway. **(A)** qRT-PCR analysis of *TGF-βR1*mRNA expression in TPC1 cells as indicated. **P <0.01 **(B)** Western blotting was used to evaluate TGF-βR1 protein expression in TPC1 cells as indicated. The average quantification obtained by densitometric analysis (Image J) of the blot of TGF-βR1 expression. Data are expressed as mean ± SEM. *P <0.05. **(C)** Western blot analysis of TGF-βR1, P-Smad2/3, and Smad2/3 protein expression in TPC1 cells as indicated. The average quantification obtained by densitometric analysis (Image J) of the blot of proteins expression. Data are expressed as mean ± SEM. *P <0.05. **(D)** Western blotting was used to evaluate E-cadherin, Vimentin, ZEB1, Snail1, TGF-βR1, and P-Smad2/3 protein expression in TPC1 cells as indicated; The average quantification obtained by densitometric analysis (Image J) of the blot of proteins expression. Data are expressed as mean ± SEM. *P <0.05. The full blot is provided in the **Supplementary File**. **(E)** CCK-8 assay was used to detect the proliferation of the indicated TPC1 cells. *P <0.05. **(F)** A wound-healing assay was applied to assess the migration ability of different TPC1 cells (LV-sh, LV-shPC- TGF-βR1). Representative images and quantification of cell counts normalized by controls are shown. *P <0.05 **(G)** Transwell invasion assays were applied to assess the motility of different TPC1 cells with a 4× objective lens. ****P <0.0001. The number of invaded cells is shown in histograms on the below.

**Figure 6:**
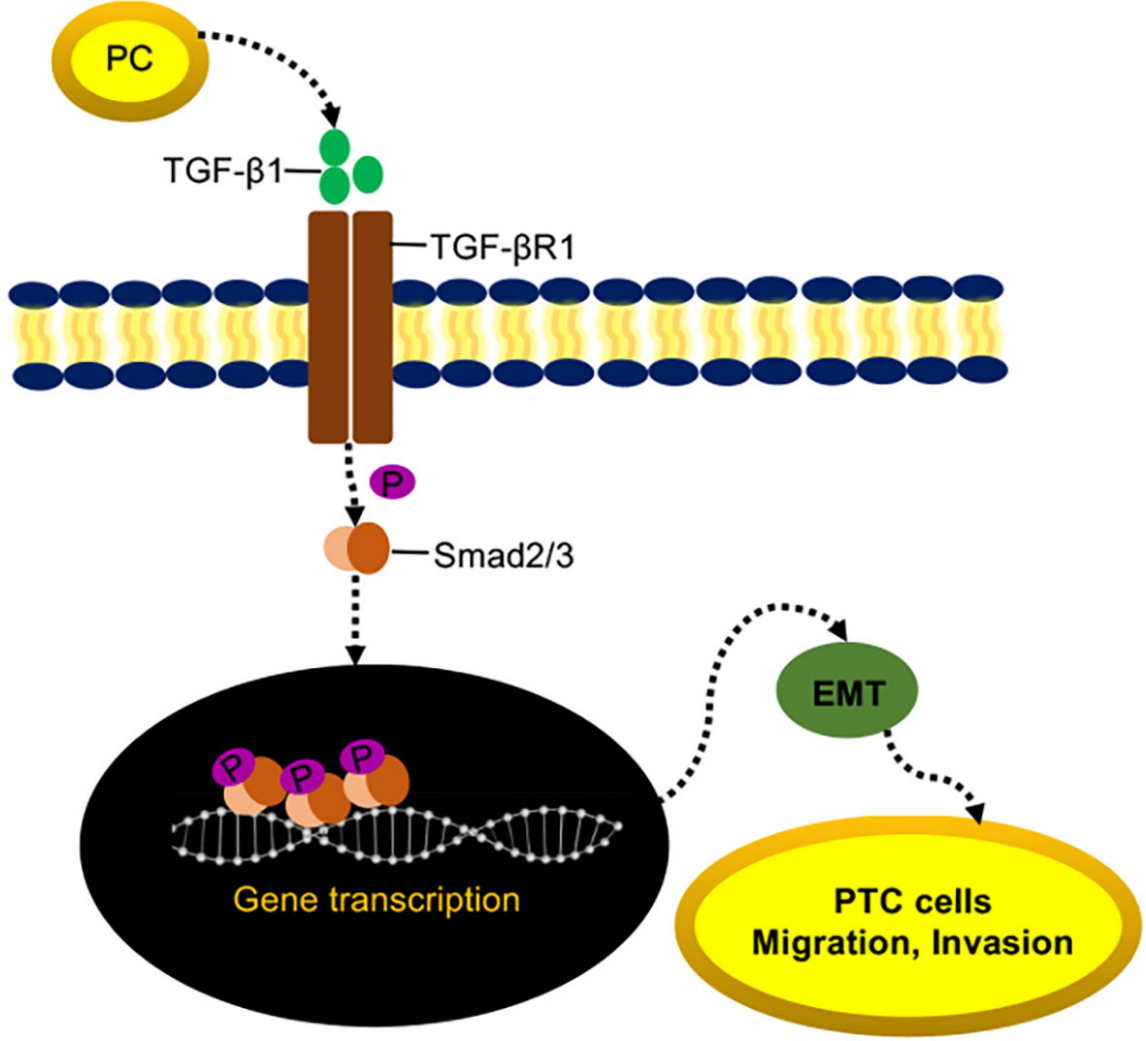
Proposed model of PC-mediated PTC migration and invasion.

Both the mRNA and protein expressions of TGF-βR1 were significantly decreased in LV-shPC cells compared to LV-NC cells (**Figures 5A, B**). Compared to those in LV-shPC cells, the proteins of ZEB1, Snali1, Vimentin, and P-Smad2/3 were upregulated and the protein level of E-cadherin was downregulated in LV-shPC-TGF-βR1 cells (**Figures 5C, D**). In addition, the proliferation, migration, and invasion abilities were significantly increased in LV-shPC-TGF-βR1 cells compared to LV-shPC cells (**Figures 5E–G**). These results suggest that PC induces PTC cells aggressiveness through the TGF-β/Smad pathway (**Figure 6**).

## Discussion

Although most PTCs tend to have “bioinert” characteristics, some show higher invasive and aggressive clinical features. Cervical lymph node metastasis is a common aggressive clinical feature, occurring in nearly half of PTC patients (39) and in 20–90% of PTMC patients (40, 41). A review of a Surveillance, Epidemiology, and End Results (SEER) database study found that cervical lymph node metastases indicate a high risk of locoregional recurrence and poor survival (42–44). Bake et al. (45) reported that PTC with lymph node metastasis group had a shorter disease-free survival period than the group without lymph node metastasis. Detection of metastasis in greater than 30% of the monitored lymph nodes is a significant independent prognostic factor in PTC (46). CLNM, representing metastasis to the sentinel cervical lymph node, is also a risk factor for recurrence (47). However, it is often difficult to recognize central lymph nodes *via* ultrasonography (48).

Molecular tests have improved the accuracy of ultrasound-guided FNA cytology (49–51), but there is no effective molecular marker for predicting lymph node metastasis. Although BRAF^V600E^ and TERT^C228T^ mutations are widely assessed in the clinic, 40% of PTCs with distant metastases are negative for BRAF or TERT genetic mutation (52). Liu et al. (53) found that the TERT^C228T^ mutation and the BRAF^V600E^ mutation led to dedifferentiation and aggressive biological behavior in THCA (54). However, the probability of the BRAF^V600E^ and TERT^C228T^ mutations occurring simultaneously in PTC is 13% (55). In addition, Ren et al. (56) reported that the coexistence of BRAF^V600E^ and TERT^C228T^ mutations has no obvious correlation with PTC lymph node metastasis. Hence, finding a new biomarker is important in the management of PTC.

Overexpression of PC is found in many human cancers (13, 57, 58). We performed bioinformatics analysis of online databases and found that the *PC* mRNA expression level was significantly higher in the PTC group than in the normal group, which was confirmed in our PTC patient surgical tumor tissues and thyroid nodule FNA wash-out fluid samples. In addition, both the mRNA and protein expression levels of PC were higher in the CLNM-positive PTC group than in the CLNM-negative group. Based on the univariate and multivariate analyses, we found that PC was a risk factor for CLNM in the PTC. Age younger than 55 was also a risk factor. This is consistent with Liu et al.’s findings (59). Factors such as extrathyroidal extension, multifocal tumors, bilateral distribution, and TNM staging were not risk factors in our study, although prior study from Feng et al. indicated that extrathyroid invasion is a risk factor (60). In Liu’s studies (61) multifocality was not risk factors either, consistently with our study. Bilateral invasion is more controversial. Yan and Xu’s studies (62, 63) showed that bilateral invasion is a risk factor, but not in the study of Zhang et al. (64).

As PC show a significant role in PTC progression and metastasis, we further explored the molecular mechanism. EMT is a typical feature in tumor metastasis. E-cadherin is an important intercellular adhesion molecule that maintains the phenotype of epithelial cells. Decreased expression of E-cadherin is an important sign of EMT. This study found that silencing PC can inhibit the proliferation, invasion and metastasis of THCA cells, upregulate the E-cadherin protein expression and decrease Vimentin, Snail1and ZEB1 protein expression. These results suggest that PC induces the EMT of PTC.

The TGF-β/Smad signaling pathway induces cancer cells immune escape, metastasis and angiogenesis by regulating EMT (65–67). The protein expression levels of P-Smad2/3 and TGF-βR1 can reflect the status of the TGF-β/Smad signal pathway, and increased protein expression of P-Smad2/3 and TGF-βR1 indicates that the TGF-β/Smad signaling pathway is activated. In contrast, decrease in P-Smad2/3 and TGF-βR1 protein expression indicates that the TGF-β/Smad signaling pathway is inhibited (68–70). This study found that silencing PC can reduce the protein expression of P-Smad2/3 and TGF-βR1 proteins in cells and thus inhibit EMT. However, the effect can be recused by overexpressing TGF-βR1, indicating that the mechanism by which silencing PC inhibits the malignant biological behavior of THCA cells may be related to inhibition of the TGF-β/Smad signaling pathway, which thereby inhibits EMT in THCA cells. We postulate that it is most likely thorough indirect action and less likely by direct binding, as PC is an intracellular enzyme. Further studies are needed to elucidate the underlying activation mechanism.

Our results showed that *PC* expression in thyroid FNA wash-out fluid may be an independent predictor for CLNM in PTC. Surgical nodal dissection should be considered in PTC patients with increased *PC* mRNA expression in the thyroid FNA wash-out fluid. This approach may improve patient prognosis and survival rates, which could be confirmed prospectively in the future study. Alternatively, active surveillance should be suitable for patients with low or no *PC* expression, which may improve the quality of life of PTC patients.

In conclusion, our study is the first to report that PC in the thyroid FNA wash-out fluid is an independent predictor for CLNM in PTC. The mechanism by which PC promotes PTC progression may be related to inducing the activation of the TGF-β/Smad signaling pathway, thereby promoting EMT in THCA cells.

## Data Availability Statement

The datasets presented in this study can be found in online repositories. The names of the repository/repositories and accession number(s) can be found in the article/**Supplementary Material**.

## Ethics Statement

This project was approved by the Ethical Committee of the Rui Jin Hospital of Shanghai JiaoTong University School of Medicine and written informed consent was obtained from all patients.

## Author Contributions

CL and LZ contributed equally to the article. CL contributed to the collection of the thyroid FNA wash-out fluid samples, RT-PCR, data analysis for the work, and drafting the manuscript. LZ contributed to the operation of ultrasound-guided fine-needle aspiration and data analysis for the work. YL and QZ contributed to the collection of the surgical tissues. YP and YZ contributed to the conception and design of the work and critically revised the manuscript. All authors contributed to the article and approved the submitted version.

## Funding

This work was supported by the National Natural Science Foundation of china (nos. 81471688, 81671720, 81801726, and 81971644), the Foundation of National Facility for Translational Medicine (Shanghai) (TMSK-2020-116), and the Shanghai Sailing Program (18YF1414300).

## Conflict of Interest

The authors declare that the research was conducted in the absence of any commercial or financial relationships that could be construed as a potential conflict of interest.

## Supplementary Material

The Supplementary Material for this article can be found onlineat: https://www.frontiersin.org/articles/10.3389/fonc.2021.643416/full#supplementary-material
