# Corrigendum: Value of Pyruvate Carboxylase in Thyroid Fine-Needle Aspiration Wash-Out Fluid for Predicting Papillary Thyroid Cancer Lymph Node Metastasis

**DOI:** 10.3389/fonc.2021.724796

**Published:** 2021-06-28

**Authors:** Chang Liu, Lu Zhang, Yang Liu, Qingqing Zhao, Yu Pan, Yifan Zhang

**Affiliations:** ^1^ Department of Nuclear Medicine, Ruijin Hospital, Shanghai Jiao Tong University School of Medicine, Shanghai, China; ^2^ Department of Ultrasound, Ruijin Hospital, Shanghai Jiao Tong University School of Medicine, Shanghai, China

**Keywords:** papillary thyroid carcinoma, pyruvate carboxylase, biopsy, fine-needle, lymphatic metastasis

There is an error in the Funding statement. The correct number for Foundation of National Facility for Translational Medicine (Shanghai) is TMSK-2020-116.

The authors apologize for this error and state that this does not change the scientific conclusions of the article in any way. The original article has been updated.

