# Supplementary material for: Value of Pyruvate Carboxylase in Thyroid Fine-Needle Aspiration Wash-Out Fluid for Predicting Papillary Thyroid Cancer Lymph Node Metastasis
Source: Front Oncol. 2021 May 17;11:643416. doi: 10.3389/fonc.2021.643416 (PMC8202284; doi:10.3389/fonc.2021.643416)
Supplement: Supplementary file 1 [file DataSheet_1.docx]

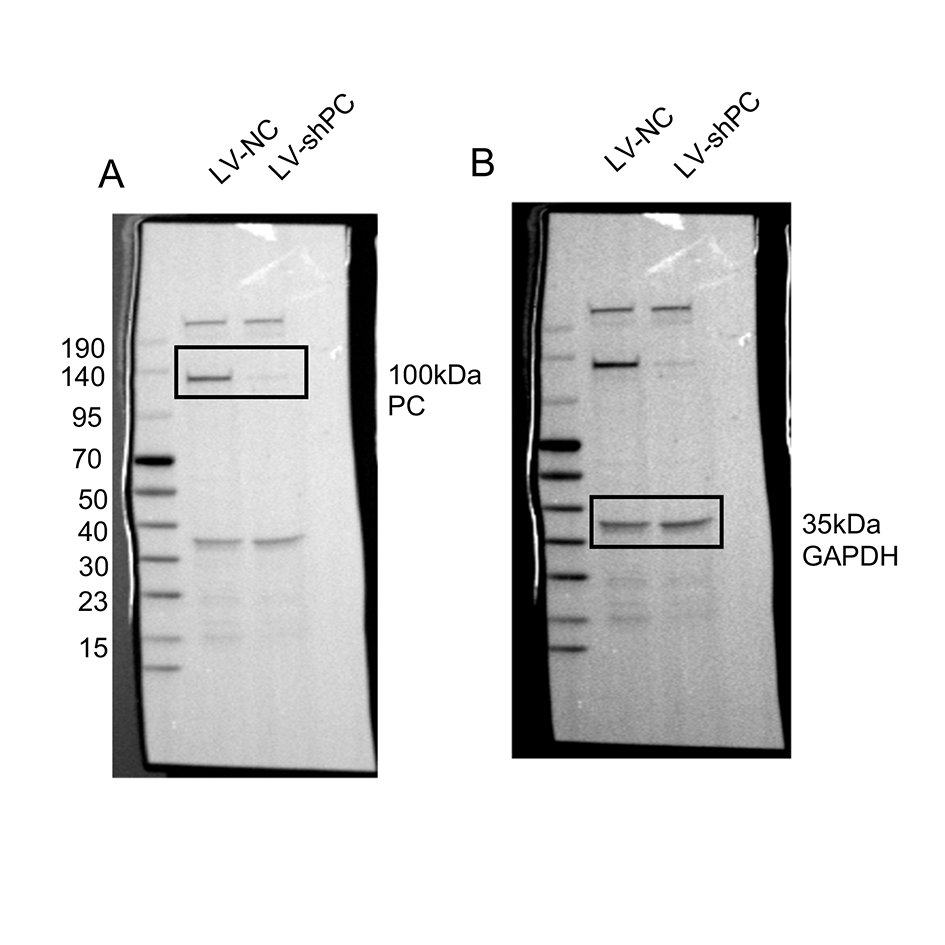


**Supplementary Figure 1**. The original, full blot for PC and GAPDH expression.


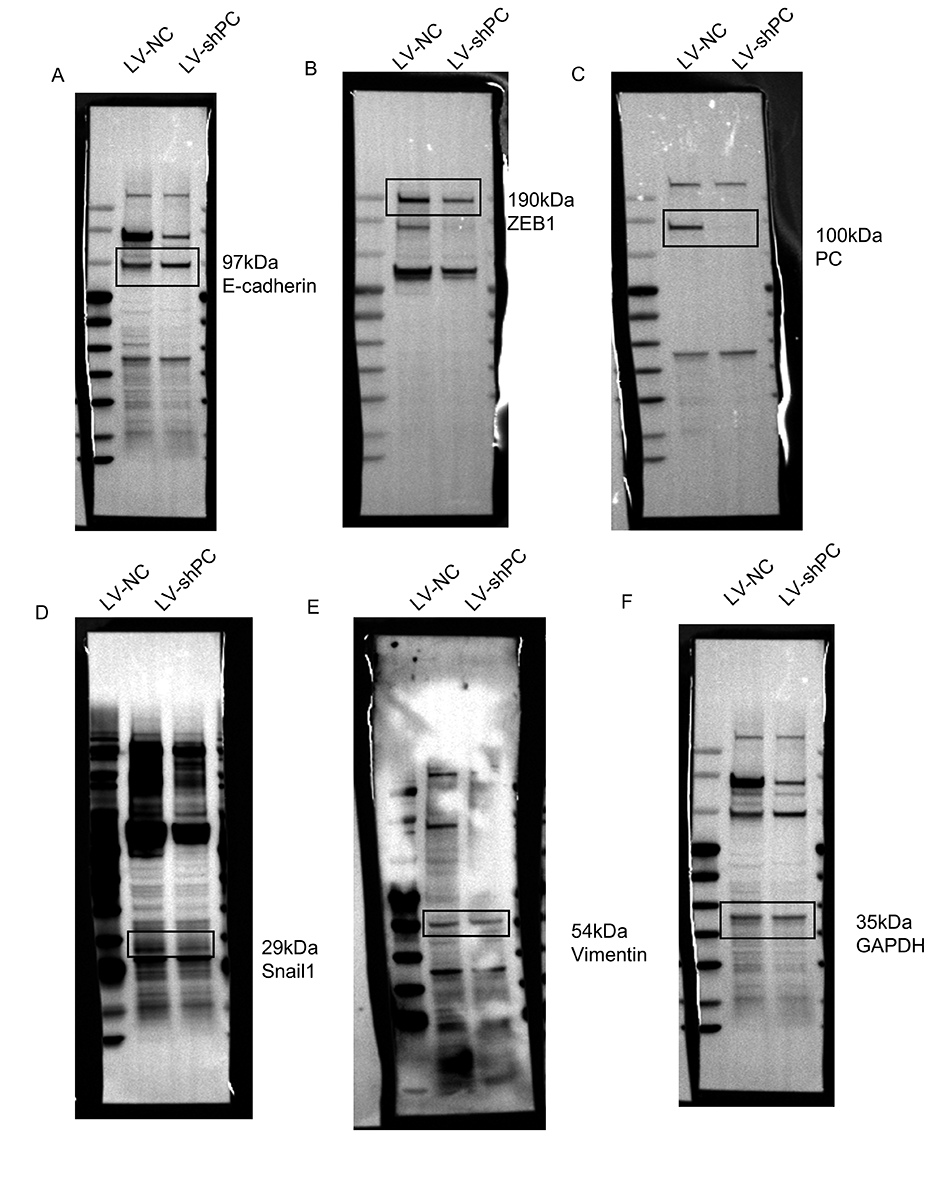


**Supplement Figure 2.** The original, full blot for E-cadherin, ZEB1, PC, Snail1, Vimentin, and GAPDH expression.


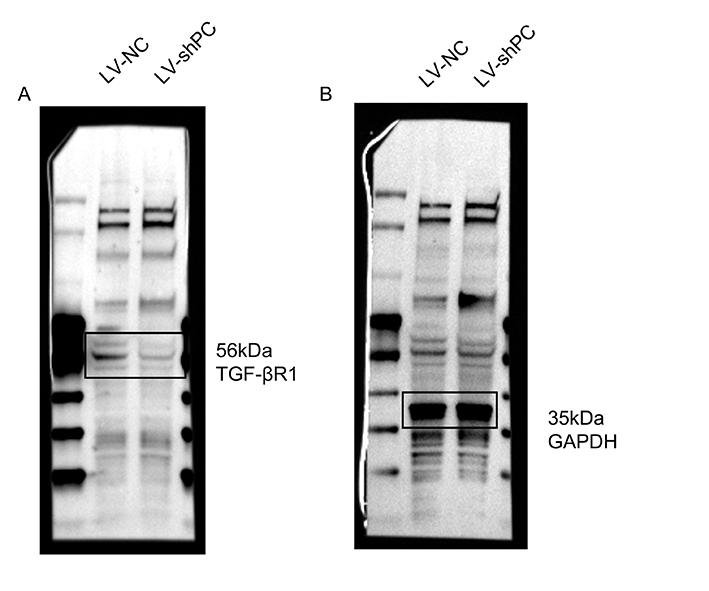


**Supplement Figure 3.** The original, full blot for TGF-βR1and GAPDH expression.


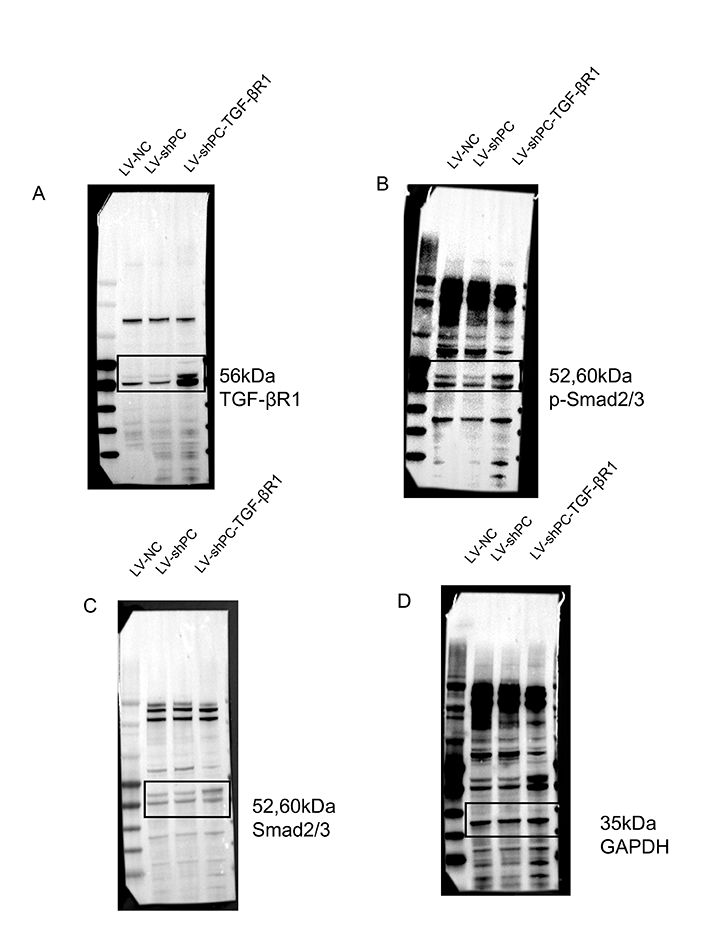


**Supplement Figure 4.** The original, full blot for TGF-βR1, p-Smad2/3, Smad2/3, and GAPDH expression.


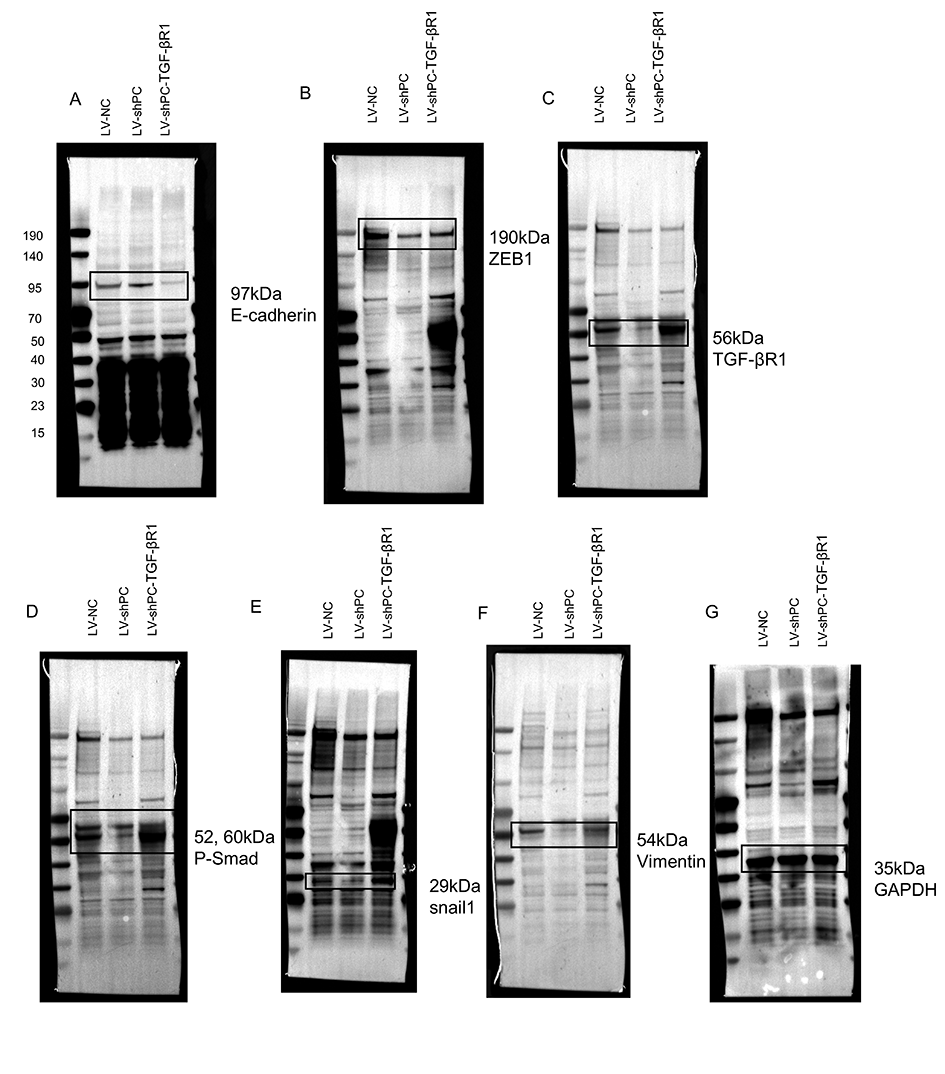


**Supplement Figure 5.** The original, full blot for E-cadherin, ZEB1, TGF-βR1, p-Smad2/3, Snail1, Vimentin, and GAPDH expression.
